# Differential detection of megakaryocytic and erythroid DNA in plasma in hematological disorders

**DOI:** 10.1038/s41525-024-00423-x

**Published:** 2024-08-05

**Authors:** W. K. Jacky Lam, Wanxia Gai, Jinyue Bai, Tommy H. C. Tam, Wai Fung Cheung, Lu Ji, Irene O. L. Tse, Amy F. C. Tsang, Maggie Z. J. Li, Peiyong Jiang, Man Fai Law, Raymond S. M. Wong, K. C. Allen Chan, Y. M. Dennis Lo

**Affiliations:** 1 Centre for Novostics, Hong Kong Science Park, Pak Shek Kok, New Territories, Hong Kong SAR, China; 2 Li Ka Shing Institute of Health Sciences, The Chinese University of Hong Kong, Shatin, New Territories, Hong Kong SAR, China; 3 Department of Chemical Pathology, Prince of Wales Hospital, The Chinese University of Hong Kong, Shatin, New Territories, Hong Kong SAR, China; 4 State Key Laboratory of Translational Oncology, Prince of Wales Hospital, The Chinese University of Hong Kong, Shatin, New Territories, Hong Kong SAR, China; 5 Department of Medicine and Therapeutics, Prince of Wales Hospital, The Chinese University of Hong Kong, Hong Kong SAR, China

**Keywords:** Epigenetics analysis, Translational research

## Abstract

The tissues of origin of plasma DNA can be revealed by methylation patterns. However, the relative DNA contributions from megakaryocytes and erythroblasts into plasma appeared inconsistent among studies. To shed light into this phenomenon, we developed droplet digital PCR (ddPCR) assays for the differential detection of contributions from these cell types in plasma based on megakaryocyte-specific and erythroblast-specific methylation markers. Megakaryocytic DNA and erythroid DNA contributed a median of 44.2% and 6.2% in healthy individuals, respectively. Patients with idiopathic thrombocytopenic purpura had a significantly higher proportion of megakaryocytic DNA in plasma compared to healthy controls (median: 59.9% versus 44.2%; *P* = 0.03). Similarly, patients with β-thalassemia were shown to have higher proportions of plasma erythroid DNA compared to healthy controls (median: 50.9% versus 6.2%) (*P* < 0.0001). Hence, the concurrent analysis of megakaryocytic and erythroid lineage-specific markers could facilitate the dissection of their relative contributions and provide information on patients with hematological disorders.

## Introduction

Analysis of plasma DNA has wide clinical applications as exemplified by non-invasive prenatal testing^1,2^ and detection of targetable mutations^3^ in precision oncology. Given the complex nature of plasma DNA released from different cell and tissue types into the circulation^4–11^, it would be of great importance to have a solid understanding of this phenomenon, thus laying the foundation for future clinical utilities. We and other researchers have devised methods to determine the tissue of origin of plasma DNA through methylation analysis^6,7,9,12^. For instance, by comparing plasma DNA methylation patterns with different tissue reference methylomes, a holistic view of the tissue contributions in plasma DNA could be achieved, named as ‘plasma DNA tissue mapping’^6^. This technology has been validated in pregnancy, transplantation, and cancer models. Zemmour et al. further expanded this technology to more than 20 tissues^13^. A more targeted approach would be to focus on the contribution from one specific cell or tissue type^9,14,15^. As an example, we previously developed erythroblast-specific markers to study erythroid DNA among healthy controls and patients with various anemic conditions by droplet digital PCR (ddPCR)^9^. Similarly, Lehmann-Werman et al. utilized pancreatic β-cell-specific and brain-specific markers to interrogate patients with diabetes mellitus and acute brain damage, respectively, by amplicon sequencing^15^.

Blood cells are known to be the major contributors to plasma DNA^8^. We revealed a substantial proportion of erythroid DNA of about 30% in plasma of healthy controls, on the basis of ddPCR assay of a methylation marker located on the ferrochelatase (*FECH*) gene^9^. However, it was recently suggested that there was a substantial contribution from megakaryocytes in the plasma DNA pool, instead of erythroid DNA contribution, based on the histone modification profiles from the chromatin immunoprecipitation (ChIP)-sequencing and whole genome bisulfite sequencing of plasma^11,12^. Therefore, in this study, we performed targeted detection of megakaryocytic and erythroid DNA in plasma of healthy individuals to investigate such a discrepancy. Also, we explored the clinical potential of the use of blood lineage specific methylation markers in patients with hematological disorders. We scrutinized the methylomes across various tissues to identify the megakaryocyte- and erythroblast-specific methylation markers, facilitating the development of ddPCR assays for targeted DNA detection. We focused on markers exhibiting differential methylation between erythroblasts and megakaryocytes. We then applied the resultant digital PCR assays in plasma DNA samples from healthy individuals and those from patients with β-thalassemia and idiopathic thrombocytopenic purpura (ITP**)**.

## Results

### Identification of megakaryocyte- and erythroblast-specific differentially methylated regions (DMRs)

**Fig. 1:**
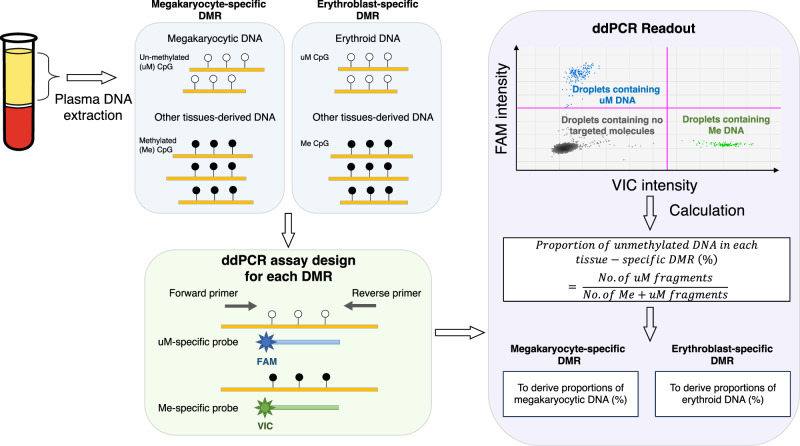
Schematic illustration of the detection of megakaryocytic and erythroid DNA through identification of cell-specific markers and digital PCR. The reference methylomes of various tissues and cells types were studied to identify the megakaryocyte- and erythroblast-specific differentially methylated regions (DMRs). We then developed a digital PCR assay to quantify the methylated and unmethylated DNA sequences each over the megakaryocyte- and erythroblast-specific DMRs, in order to infer the proportions of megakaryocytic DNA and erythroid DNA.

A schematic illustration of the identification of megakaryocyte- and erythroblast-specific differentially methylated regions (DMRs) and the design of ddPCR assays is shown in Fig. 1. First, the genome-wide methylation profiles of erythroblasts, megakaryocytes and other types of blood cells (neutrophils, B cells, and T cells) and tissues (adipose tissues, adrenal gland, colon, esophagus, liver, lung, pancreas and small intestines) were first retrieved from public datasets available on the Roadmap Epigenomics^16^, the Encyclopedia of DNA Elements (ENCODE)^17^ project and Blueprint Epigenome^18^. The methylation densities, defined as the CpG loci being methylated, of all CpG sites on autosomes were systematically compared to identify the DMRs. Details of the retrieval of reference methylomes and data processing for the development of DMRs were described in the Methods section. The megakaryocyte- and erythroblast-specific DMRs were determined using the following criteria:

1. In defining the megakaryocyte-specific and erythroblast-specific DMRs, we would identify a hypomethylated region specific to the target cell types (i.e. megakaryocytes or erythroblasts), in which the methylation densities of the CpG sites in all the other cell or tissue types over the CpG sites in that region exceeded 75%.
2. Multiple CpG sites would be included in the DMR if possible, to improve the signal-to-noise ratio. Also, the selected CpG sites that were located within close genomic proximity would have the comparative advantage for the analysis in plasma DNA molecules, given that plasma DNA is short in nature with a modal size of about 160 bp^2^. In addition, to facilitate the probe design, DMR with at least 3 CpG sites within 20 bp would be considered.

### Megakaryocyte- and erythroblast-specific methylation markers

Based on the above-mentioned criteria, we have identified a number of DMRs across the whole genome and selected one megakaryocyte-specific DMR and one erythroblast-specific DMR with the largest difference in the methylation densities between the target cell type versus the other cell types (Supplementary Fig. 1). Other candidate DMRs are shown in Supplementary Fig. 2. There is a substantial difference in the methylation levels between erythroblast and megakaryocyte over these two selected DMRs to avoid the cross detection of erythroid and megakaryocytic DNA. The megakaryocyte-specific methylation marker region, named as Mk-1, is located on the paralemmin 2 and A-kinase anchoring protein 2 (*PALM2AKAP2*) gene on chromosome 9 (Supplementary Fig. 1a). A-kinase anchoring proteins have been found in platelets, but their roles in platelet activation are largely unknown^19^. The erythroblast-specific methylation marker has been reported by us before and named as Ery-1^9^. The Ery-1 marker is located on chromosome 12 and the genomic region associated with this DMR has not been identified with any annotated gene^9^ (Supplementary Fig. 1b).

In addition, we have reviewed the methylation densities of megakaryocytes, erythroblasts and other cells and tissues types in the previously reported erythroid methylation marker located in the intronic region of the ferrochelatase (*FECH*) gene^9^. As shown in Supplementary Fig. 3a, the 4 CpG sites within this marker were hypomethylated (less than 20%) for both megakaryocytes and erythroblasts. The other previously reported erythroid marker Ery-2^9^, similar to Ery-1, was shown to be specific to erythroblasts when only erythroblasts but not megakaryocytes or other tissue or cell types (except adrenal gland) were hypomethylated in this DMR (Supplementary Fig. 3b).

### Detection of megakaryocytic and erythroid DNA through methylation analysis by digital PCR

To deduce the proportions of megakaryocytic and erythroid DNA in plasma through methylation, we have developed a digital PCR assay to quantify the methylated and unmethylated DNA sequences each over the megakaryocyte- and erythroblast-specific DMRs (Fig. 1). Details of the primers and probes of both digital PCR assays are described in the Methods section and Supplementary Table 1. The probes were designed to target fully methylated (all 3 CpG sites being methylated) and fully unmethylated (all 3 CpG sites being unmethylated) DNA sequences over these DMRs after bisulfite treatment of plasma samples. The analytical specificity of these assays was confirmed with the use of synthetic fully methylated and fully unmethylated DNA sequences as positive and negative controls and the details were described in both the Methods section and Supplementary Fig. 4.

**Fig. 2:**
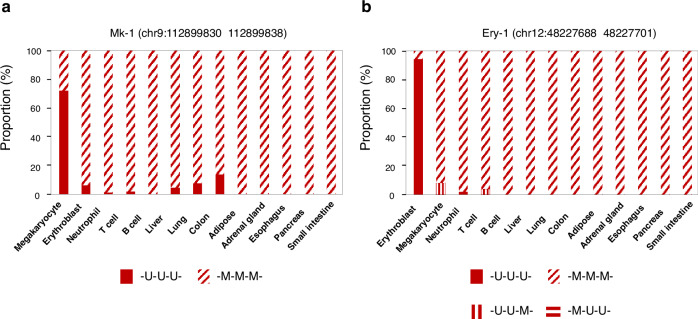
Relative fractions of unmethylated haplotypes and methylated haplotypes in the differentially methylated regions (DMRs) of megakaryocytes and erythroblasts for the adjustment of the calculation of megakaryocytic and erythroid DNA contributions. The relative fractions in the different cell types are shown. The unmethylated CpG is denoted as U and the methylated CpG is denoted as M. **a** Megakaryocyte-specific DMR. The relative fractions of the -U-U-U- and -M-M-M- haplotypes in the different cell types are shown. **b** Erythroblast-specific DMR. The relative fractions of the -U-U-U-, -U-U-M-, -M-U-U- and -M-M-M- haplotypes in the different cell types are shown.

Since we applied digital PCR for analysis of methylation in the corresponding DMRs to infer the proportions of megakaryocytic DNA and erythroid DNA, we first studied the relative fractions of unmethylated and methylated DNA haplotypes in the reference methylome data of the different cell types. First, we performed an experiment to determine the reactivity between DNA sequences bearing all the 8 different methylation patterns of the 3 target CpG sites (i.e. -U-U-U-, -M-M-M- and 6 mosaic patterns including -U-U-M-, -U-M-U-, -M-U-U-, -M-M-U-, -M-U-M-, -U-M-M-, where U denotes unmethylated CpG and M denotes methylated CpG) and the probes (which were designed to target fully methylated (-M-M-M-) and fully unmethylated (-U-U-U-) sequences). The results showed that, in the Mk-1-based digital PCR analysis, none of the DNA of mosaic methylation patterns yielded a positive signal above the threshold (Supplementary Fig. 5). Therefore, the subsequent calculation of megakaryocytic DNA contribution would be adjusted according to the relative fractions of fully unmethylated (-U-U-U-) haplotypes versus fully methylated (-M-M-M-) haplotypes only in the different cell types (Fig. 2a). In the Ery-1 marker-based digital PCR analysis, DNA sequences of the -U-U-M- and -M-U-U- patterns also yielded positive signals (i.e. above the pre-defined threshold) with the probe targeting fully unmethylated (-U-U-U-) DNA (Supplementary Fig. 6). Therefore, the subsequent calculation of erythroid DNA contribution would be adjusted according to the relative fractions of -U-U-M-, -M-U-U- and -U-U-U- haplotypes versus fully methylated (-M-M-M-) haplotypes in the different cell types (Fig. 2b). The details of the experiment were described in the Methods section.

For the megakaryocytic-specific DMR, proportion of unmethylated DNA sequences measured by the corresponding assay would include predominantly proportion of unmethylated DNA from megakaryocytes and a minority from other cell types. Similarly, for the erythroblast-specific DMR, proportion of unmethylated DNA sequences measured by this assay would include predominantly that from erythroblasts and also a minority from other cell types or tissues. Therefore, the proportions of megakaryocytic (MK%) and erythroid DNA (Ery%) in a biological sample would then be derived as:

Let MK% be the proportion of megakaryocytic DNA in plasma,

$$\begin{array}{l}Proportion\,of\,unmethylated\,DNA\,sequences\,in\,the\,megakaryocytic\,DMR\,\\ measured\,by\,digital\,PCR\,analysis =Proportion\,of\,unmethylated\,megakaryocytic\,DNA\\\,+Proportion\,of\,unmethylated\,DNA\,contributed\,by\,other\,cell\,types\\ =MK \% \,\times \,relative\,fraction\,of\,fully\,unmethylated\,haplotype\,\\ over\,fully\,unmethylated\,and\,methylated\,haplotypes\,for\,megakaryocytes\,in\,the\,DMR\\ +(1-MK \% )\,\times mean\,relative\,fraction\,of\,fully\,unmethylated\\ \,haplotype\,over\,fully\,unmethylated\\ \,and\,methylated\,haplotypes\,for\,other\,cell\,types\,in\,the\,DMR,\end{array}$$

where the fraction of fully unmethylated DNA haplotype (i.e. -U-U-U-) over fully unmethylated (i.e. -U-U-U-) and methylated (i.e. -M-M-M-) haplotypes of megakaryocyte and the fractions of other cell types (the mean fraction for the other 12 cell types were used in the calculation) within this megakaryocytic DMR (Fig. 2a) were determined based on the reference methylome data and described in the Methods section.

Similarly, let Ery% be the proportion of erythroid DNA,

$$\begin{array}{l}Proportion\,of\,unmethylated\,DNA\,sequences\,in\,the\,erythroblastic\,DMR\,\\ measured\,by\,digital\,PCR\,analysis\\ =Proportion\,of\,unmethylated\,erythroid\,DNA\\+Proportion\,of\,unmethylated\,DNA\,contributed\,by\,other\,cell\,types\\ =Ery \% \,\times \,relative\,fraction\,of\,unmethylated\,{\rm{haplotypes}}\,\\ over\,unmethylated\,and\,fully\,methylated\,haplotypes\\ for\,erythroblasts\,in\,the\,DMR\\ +(1-Ery \% )\,\times \,mean\,relative\,fraction\,of\,unmethylated\,haplotypes\,\\ over\,unmethylated\,and\,fully\,methylated\,haplotypes\,for\,other\,cell\,types\,in\,the\,DMR,\end{array}$$

where the relative fraction of unmethylated DNA haplotypes (including -U-U-U-, -U-U-M- and -M-U-U-) over unmethylated and fully methylated (i.e. -M-M-M-) haplotypes of erythroblast and the fractions of other cell types (the mean fraction for the other 12 cell types were used in the calculation) within this erythroblastic DMR (Fig. 2b) were determined based on the reference methylome data and described in the Methods section.

### Detection of plasma erythroid and megakaryocytic DNA in healthy subjects

**Fig. 3:**
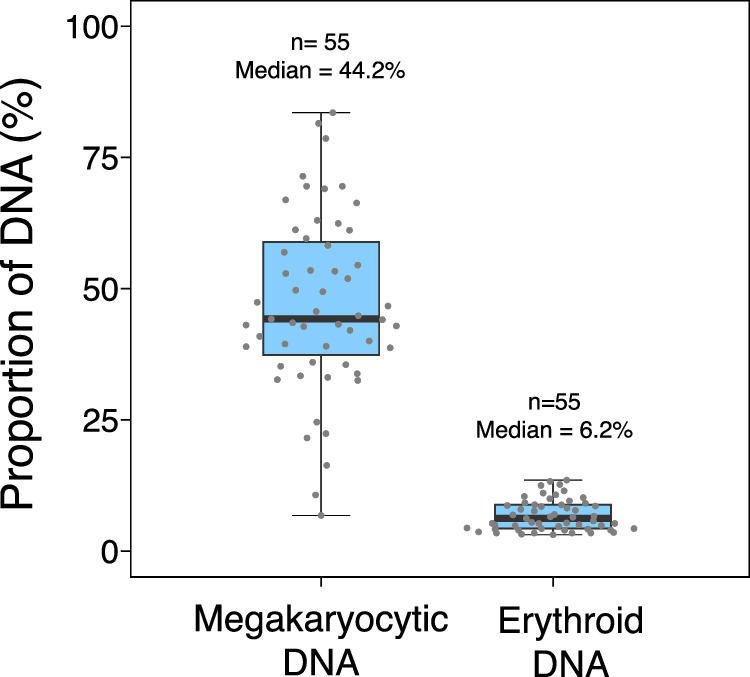
Proportions of megakaryocytic and erythroid DNA in the plasma of healthy subjects. Each dot represents the proportions of megakaryocytic and erythroid DNA in the healthy subjects. The lower and upper box limits indicate the 25^th^ and 75^th^ percentiles.

We simultaneously performed digital PCR analyses targeting the Mk-1 methylation marker and the Ery-1 marker on plasma samples from 55 healthy subjects. The median age was 57 years old (range: 16–70). The detailed demographic data of the healthy subjects were shown in Supplementary Table 2. Through the digital PCR analysis, the median proportion of megakaryocytic DNA was 44.2% (interquartile range: 37.4%–58.9%). The median proportion of erythroid DNA was 6.2% (interquartile range: 4.3%–8.8%) (Fig. 3). The results suggested that megakaryocytic DNA contributed a substantial proportion to the plasma DNA pool in healthy individuals. We did not observe any correlation between the megakaryocytic DNA proportions and age (*r* = −0.13, *P* = 0.35, Pearson correlation), or erythroid DNA and age (*r* = 0.1, *P* = 0.46, Pearson correlation) (Supplementary Fig. 7). There was no difference in the proportions of megakaryocytic and erythroid DNA between the male and female control subjects (*P* = 0.28 for megakaryocytic DNA and *P* = 0.55 for erythroid DNA) (Supplementary Fig. 8).

**Fig. 4:**
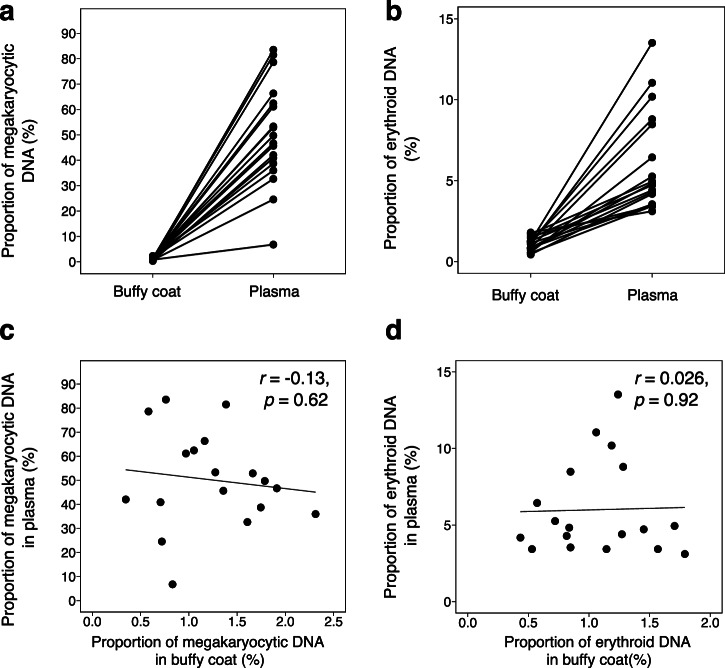
Proportions of megakaryocytic and erythroid DNA in the paired plasma and buffy coat of healthy subjects. **a** Proportions of megakaryocytic DNA in the paired plasma and buffy coat samples of healthy subjects. **b** Proportions of erythroid DNA in the paired plasma and buffy coat samples of healthy subjects. **c** Correlation between the proportions of megakaryocytic DNA detected in the plasma and the proportions in the paired buffy coat samples of healthy subjects. No correlation was observed. **d** Correlation between the proportions of erythroid DNA detected in the plasma and the proportions in the paired buffy coat samples of healthy subjects. No correlation was observed.

In addition, we have studied if there exists any correlation between the proportions of megakaryocytic DNA or erythroid DNA measured in plasma and those measured in buffy coat. We measured the proportions of megakaryocytic and erythroid DNA in the paired buffy coat samples of some (18 out of 55) healthy subjects (those with available samples). The proportion of megakaryocytic DNA measured in buffy coat DNA (median: 1.2%; interquartile range: 0.8%–1.7%) was significantly lower than that in plasma (median: 48.2%; interquartile range: 39.3% – 62.1%) (*P* < 0.0001, Mann-Whitney *U* test) among the healthy individuals (Fig. 4a). Similarly, the proportion of erythroid DNA measured in buffy coat DNA (median: 1.1%; interquartile range: 0.8%–1.3%) was also significantly lower than that measured in plasma (median: 4.8%; interquartile range: 3.7%–8.0%) (*P* < 0.0001, Mann-Whitney *U* test) (Fig. 4b). No correlation was found between the proportion of megakaryocytic DNA in plasma and that in buffy coat (*r* = −0.13, *P* = 0.62, Pearson correlation) (Fig. 4c), or between the proportion of erythroid DNA in plasma and that in buffy coat (*r* = 0.026, *P* = 0.92, Pearson correlation) (Fig. 4d). These findings suggested that both the circulating megakaryocytic and erythroid DNA molecules in plasma were unlikely to be derived from circulating erythroblasts and megakaryocytes in the peripheral blood.

### Plasma DNA analysis in β-thalassemia major

**Fig. 5:**
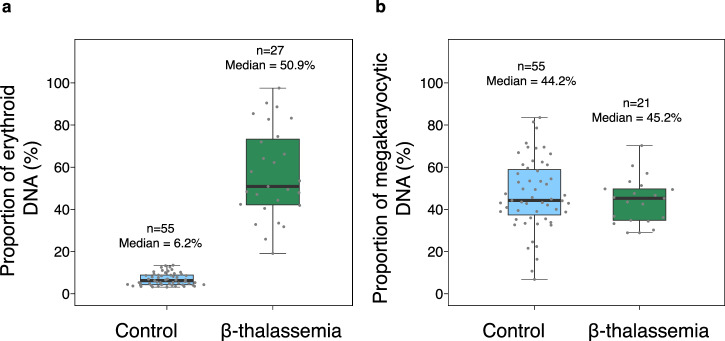
Proportions of erythroid and megakaryocytic DNA in the plasma of patients with β-thalassemia major and healthy controls. **a** Proportions of erythroid DNA in the patients with β-thalassemia major and healthy subjects. The lower and upper box limits indicate the 25^th^ and 75^th^ percentiles. **b** Proportions of megakaryocytic DNA in the patients with β-thalassemia major and healthy subjects. The lower and upper box limits indicate the 25^th^ and 75^th^ percentiles.

We performed digital PCR that targeted the Ery-1 marker in plasma of 27 patients with β-thalassemia major. The detailed demographic data of these patients were shown in Supplementary Table 2. Patients with β-thalassemia had significantly higher concentration of total plasma DNA (as measured by Qubit Fluorometer, ThermoFisher) than the control subjects (*P* < 0.0001, Mann-Whitney *U* test) (Supplementary Fig. 9). Among these patients with β-thalassemia major, the median proportion of erythroid DNA was shown to be 50.9% (interquartile range: 42.1%–73.3%), which was significantly higher than that among the healthy subjects (median: 6.2%; interquartile: 4.3%–8.8%) (*P* < 0.0001, Mann-Whitney *U* test) (Fig. 5a). The erythroid DNA detected in plasma is hypothesised to be derived from erythroblasts in the bone marrow. With such a hypothesis, the observation of higher proportion of erythroid DNA in plasma of β-thalassemia major patients could imply higher number of erythroblasts in the bone marrow. Such result of a higher proportion of erythroid DNA in plasma was compatible with the pathophysiological features of β-thalassemia^20^ because there is an increased but ineffective erythropoiesis as a result of the imbalance in α- and non-α-globin chains and premature destruction of red cell precursors. Concomitantly, we have analyzed the Mk-1 methylation by digital PCR in these patients. There was no significant difference in the proportion of megakaryocytic DNA deduced by Mk-1 methylation between patients with β-thalassemia major (median: 45.2%; interquartile range: 34.8%–49.7%) and healthy subjects (median: 44.2%; interquartile range: 37.4%–58.9%) (*P* = 0.46, Mann-Whitney *U* test) (Fig. 5b).

### Plasma DNA analysis in idiopathic thrombocytopenic purpura

**Fig. 6:**
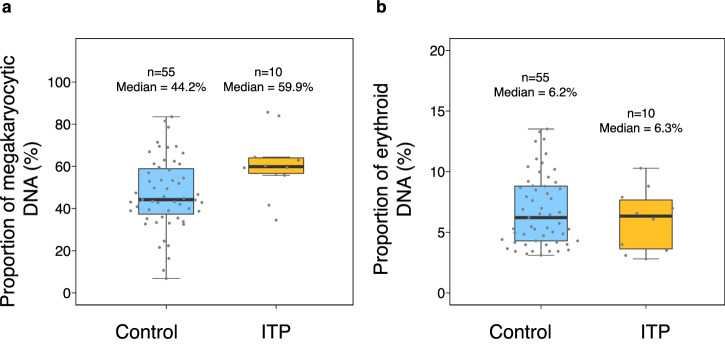
Proportions of megakaryocytic and erythroid DNA in the plasma of patients with idiopathic thrombocytopenic purpura (ITP) and healthy controls. **a** Proportions of megakaryocytic DNA in the patients with ITP and healthy subjects. The lower and upper box limits indicate the 25^th^ and 75^th^ percentiles. **b** Proportions of erythroid DNA in the patients with ITP and healthy subjects. The lower and upper box limits indicate the 25^th^ and 75^th^ percentiles.

We analyzed the proportion of megakaryocytic DNA in plasma of 10 patients with idiopathic thrombocytopenic purpura (ITP) with the digital PCR assay that targeted the MK-1 methylation marker. The demographic data of the patients were stated in the Supplementary Table 2. All patients had previous bone marrow biopsies showing hyperplasia of megakaryocytes. There was no significant difference in the total plasma DNA concentration (as measured by Qubit Fluorometer, ThermoFisher) between ITP patients and control subjects (*P* = 0.34, Mann-Whitney *U* test) (Supplementary Fig. 9). Their median proportion of megakaryocytic DNA (median: 59.9%, interquartile range: 56.7%–64.0%) was significantly higher than that detected among the healthy subjects (median: 44.2%; interquartile range: 37.4%–58.9%) (*P* = 0.03, Mann-Whitney *U* test) (Fig. 6a). The higher proportion of megakaryocytic DNA in plasma of patients with ITP is consistent with the compensatory mechanism of increased platelet production from megakaryocytes in response to the peripheral destruction of platelets. We have also studied the Ery-1 methylation in the plasma DNA of these patients with ITP. There was no significant difference in the proportion of erythroid DNA between patients with ITP (median:6.3%; interquartile range: 3.6%–7.7%) and healthy subjects (median: 6.2%; interquartile range: 4.3%–8.8%) (*P* = 0.41, Mann-Whitney *U* test) (Fig. 6b).

## Discussion

The core objective of this study was to analyze DNA of erythroid and megakaryocytic origins in plasma. Therefore, we have developed a megakaryocytic methylation marker, together with our previously developed erythroid marker^9^, to interrogate the contributions of megakaryocytic and erythroid DNA to the plasma DNA pool in healthy individuals. A majority of plasma DNA had been postulated to be derived from cells of the haemopoietic system given the close proximity of blood cells to plasma. Early evidence came from the analysis of the sex-mismatched bone marrow transplant model^8^. Among female bone marrow recipients from male donors, we have shown that there was a substantial proportion (up to 60%) of Y-chromosome DNA, as a surrogate of DNA of hemopoietic origin, in the plasma^8^. While such analysis in the bone marrow transplant model is based on the genotypic differences between donors and recipients, we observed similar findings through methylation analysis of plasma DNA in normal individuals. We have previously developed the ‘plasma DNA tissue mapping’^6^ technology to deconvolute the tissue and cell contributions of plasma DNA based on their differential methylation profiles. Using this methylation-based technology, blood cells were again shown to be a major contributor to plasma DNA. The expanding knowledge on the methylation of different tissue and cell types from international collaborative projects such as the Roadmap Epigenomics^16^ and the ENCODE projects^17^ has helped tease out the plasma DNA composition in healthy and disease conditions. We have previously established erythroid methylation markers to study the erythroid DNA contribution in plasma by digital PCR^9^. Along the same line, we now developed the megakaryocytic methylation marker, enabling the evaluation of the megakaryocytic DNA contribution in plasma.

Using the megakaryocytic methylation marker identified in this study, we have shown that there was a substantial proportion of megakaryocytic DNA (about 44.2%) in the plasma of healthy subjects. This finding is consistent with the recent analyses through chromatin immunoprecipitation-sequencing (ChIP-seq)^11^ and bisulfite sequencing^12,21^ of plasma DNA. Regarding the erythroid DNA contribution, we have identified three erythroid methylation markers in our previous study^9^. Digital PCR detection based on one of the erythroid markers, which was associated with the *FECH* gene on chromosome 18, might lead to cross-detection with megakaryocytic DNA and therefore over-estimation of erythroid DNA in plasma^9^. This point was evidenced by the fact that both erythroblasts and megakaryocytes were later found to exhibit similar hypomethylation within this region. Similarly, Moss et al. appeared to have over-estimated the erythroid DNA contribution using the methylation microarray^7^, possibly due to the inclusion of marker regions that show similar methylation patterns between erythroblast and megakaryocyte and later re-analyzed with cell-specific regions^21^. Indeed, erythroblasts and megakaryocytes could exhibit similar methylation profiles as they share the same progenitors (i.e. megakaryocytic-erythroid progenitors). Therefore, we have used another reported erythroid marker (named as Ery-1)^9^, which clearly shows differential methylation between erythroblasts and megakaryocytes, to re-evaluate the erythroid DNA contribution in the plasma DNA pool. Based on the methylation analysis on this erythroid marker, erythroid DNA was shown to be present in plasma with a median proportion of 6.2%. These results support the presence of erythroid DNA in plasma of healthy individuals, though at a lower proportion than previously estimated.

Our digital PCR analysis demonstrated higher proportions of megakaryocytic DNA in the plasma of patients with ITP compared to healthy subjects, which was consistent with the disease pathophysiology. These results suggest that quantitative analysis of megakaryocytic DNA in plasma could reflect megakaryopoietic activity in the bone marrow. Such analysis might potentially serve as an adjunct to bone marrow biopsy for evaluation of the megakaryocyte status in platelet-related disorders. In addition, the rise in the proportion of megakaryocytic DNA as deduced by this megakaryocytic marker was only observed in the ITP patients but not in β-thalassemia major patients. Future studies could include the analysis of plasma megakaryocytic DNA in a larger cohort of healthy subjects and patients with ITP and other platelet-related disorders. The clinical utility of plasma megakaryocytic DNA measurement for differential diagnosis, prognostication and monitoring of treatment response could be further explored.

In summary, through defining megakaryocyte- and erythroblast-specific methylation markers, we have demonstrated the presence of megakaryocytic and erythroid DNA in plasma. Megakaryocytic DNA was shown to contribute substantially to the plasma DNA pool, while erythroid DNA had a relatively lower contribution. The proposed analysis of megakaryocytic and erythroid markers demonstrates the diagnostic potential in patients with the corresponding blood lineage disorders.

## Methods

### Retrieval of reference methylomes and data processing

The reference methylomes of megakaryocytes, erythroblasts and other blood cells and tissue types (neutrophils, B cells, and T cells, adipose, adrenal gland, colon, esophagus, liver, lung, pancreas and small intestines) were retrieved from publicly available resources including Roadmap Epigenomics^16^, the Encyclopedia of DNA Elements (ENCODE)^17^ project and Blueprint Epigenome^18^. The reference methylomes of all the available samples/ data files at the time of marker development for each cell or tissue type have been used and the list of reference methylomes were provided in the Supplementary Table 3. To elaborate, we first downloaded the raw bisulfite sequencing data and then performed data processing and alignment with our previously developed pipeline, Methy-Pipe^22^. After that we pooled the sequencing data and obtained the genome-wide methylation of all the available samples for each cell or tissue type at base pair resolution, which was then used for identification of megakaryocyte- and erythroblast-specific DMRs.

As mentioned, the proportions of megakaryocytic and erythroid DNA were adjusted for the baseline methylation of megakaryocytes, erythroblasts and other cell types in the corresponding DMRs. The relative fractions of unmethylated and methylated DNA haplotypes of the different cell and tissue types were obtained from the reference methylomes. We performed an experiment to determine the reactivity between DNA sequences bearing all the 8 different methylation patterns of the 3 target CpG sites (i.e., -U-U-U-, -M-M-M-, and 6 mosaic patterns including -U-U-M-, -U-M-U-, -M-U-U-, -M-M-U-, -M-U-M- and -U-M-M-) and the probes (which were designed to target fully methylated -M-M-M- and fully unmethylated -U-U-U- sequences). In detail, we ordered synthetic DNA oligos of the 8 methylation patterns (Integrated DNA Technologies, Iowa) and analyzed with the two digital PCR assays targeting the Ery-1 and Mk-1 markers (Supplementary Figs. 5 and 6). In the Mk-1-based digital PCR analysis, none of the DNA of mosaic methylation patterns yielded a positive signal above the threshold (Supplementary Fig. 5). Therefore, the subsequent calculation of megakaryocytic DNA contribution would be adjusted according to the relative fractions of fully unmethylated (-U-U-U-) haplotypes versus fully methylated (-M-M-M-) haplotypes only in the different cell types (Fig. 2a). In the Ery-1 marker-based digital PCR analysis, DNA sequences of the -U-U-M- and -M-U-U- patterns also, but not other mosaic patterns, yielded positive signals with the probe targeting fully unmethylated DNA (Supplementary Fig. 6). Therefore, the subsequent calculation of erythroid DNA contribution would be adjusted according to the relative fractions of -U-U-M-, -M-U-U- and -U-U-U- haplotypes versus fully methylated (-M-M-M-) haplotypes in the different cell types (Fig. 2b).

In the deduction of megakaryocytic DNA (MK%) through the Mk-1-based analysis, the fraction of fully unmethylated (-U-U-U-) DNA haplotype over fully unmethylated (-U-U-U-) and methylated (-M-M-M-) haplotypes of megakaryocytes was 72.2% and the mean fraction of the other 12 cell types was 3.0%. In the deduction of erythroid DNA (Ery%) through Ery-1-based analysis, the relative fraction of unmethylated (-U-U-U-, -U-U-M-, -M-U-U-) DNA haplotype to unmethylated and fully methylated (-M-M-M-) haplotypes of erythroblasts was 94.4% and the mean fraction of the other 12 cell types was 1.2%.

### Subject recruitment

To study the erythroid and megakaryocytic DNA in plasma, we have recruited healthy subjects and patients with β-thalassemia and ITP from the Department of Chemical Pathology and the Division of Haematology of the Department of Medicine and Therapeutics of the Prince of Wales Hospital of Hong Kong, respectively. All subjects gave written informed consent and the study was approved by the Joint Chinese University of Hong Kong-Hospital Authority New Territories East Cluster Clinical Research Ethics Committee under the Declaration of Helsinki.

### Sample collection, plasma DNA extraction

For each recruited subjects, peripheral venous blood was collected into an ethylenediaminetetraacetic acid (EDTA)-containing tube. Plasma isolation was performed within 6 h of sample collection with a protocol consisting of a first centrifugation at 1600 g for 10 min followed by re-centrifugation of the plasma portion at 16,000 g for another 10 min. Plasma DNA extraction was done with the QIAamp Circulating Nucleic Acid Kit (Qiagen).

### Bisulfite conversion of DNA

Plasma DNA was subjected to two rounds of bisulfite treatment using Epitect Plus Bisulfite Kit (Qiagen) according to the manufacturer’s instructions.

### Droplet digital PCR analysis

The primers and probes design for the assays are illustrated in the Supplementary Table 1. Each sample was tested in duplicate for each digital PCR assay after bisulfite treatment. A total volume of 20 μL of each reaction mix was prepared, containing 8 μL of template DNA, a final concentration of 900 nM of the forward primers, 900 nM of the reverse primer and 250 nM of the probe. The reaction mix was then subjected to a BioRad QX200/QX100 Droplet Generator for droplets generation according to the manufacturer’s instructions. The droplets were transferred into a clean 96-well plate followed by thermal cycling using the following condition for the Ery-1 and MK-1 assays: 95 °C × 10 min (1 cycle), 40 cycles of 94 °C × 15 s and 61 °C (Ery-1) or 58 °C (MK-1) × 1 min, 98 °C × 10 min (1 cycle), followed by a 12 °C hold step. After the PCR, the droplets readings were carried out by a BioRad QX200/100 reader and the results were analyzed using the QuantaSoft (version 1.7) software.

The synthetic, fully methylated sequences from the CpGenome Human Methylated DNA (EMD Millipore) and the fully unmethylated sequences from the EpiTect Unmethylated Human Control DNA (Qiagen) were used to confirm the analytical specificity of the digital PCR assays based on the megakaryocyte- and erythroblast-specific DMRs (Supplemental Fig. 4). The CpGenome Human Methylated DNA was purified from HCT116 DKO cells followed by enzymatic methylation of all CpG nucleotides using M.SssI methyltransferase. The fully methylated and fully unmethylated DNA sequences were run on the same plate as positive and negative controls. The cut-off values for positive fluorescence signals were determined with reference to the controls. The numbers of methylated and unmethylated DNA sequences in each sample was calculated using combined counts from duplicate wells followed by Poisson correction^23^.

### Supplementary information

Supplementary Figures 1-9

Supplementary Tables 1-3

## Data Availability

All data generated or analyzed during this study are included in this published article.
