# Supplementary material for: Differential detection of megakaryocytic and erythroid DNA in plasma in hematological disorders
Source: NPJ Genom Med. 2024 Aug 5;9:39. doi: 10.1038/s41525-024-00423-x (PMC11300836; doi:10.1038/s41525-024-00423-x)
Supplement: Supplementary file 1 — Supplementary Figures 1-9 [file 41525_2024_423_MOESM1_ESM.pdf]

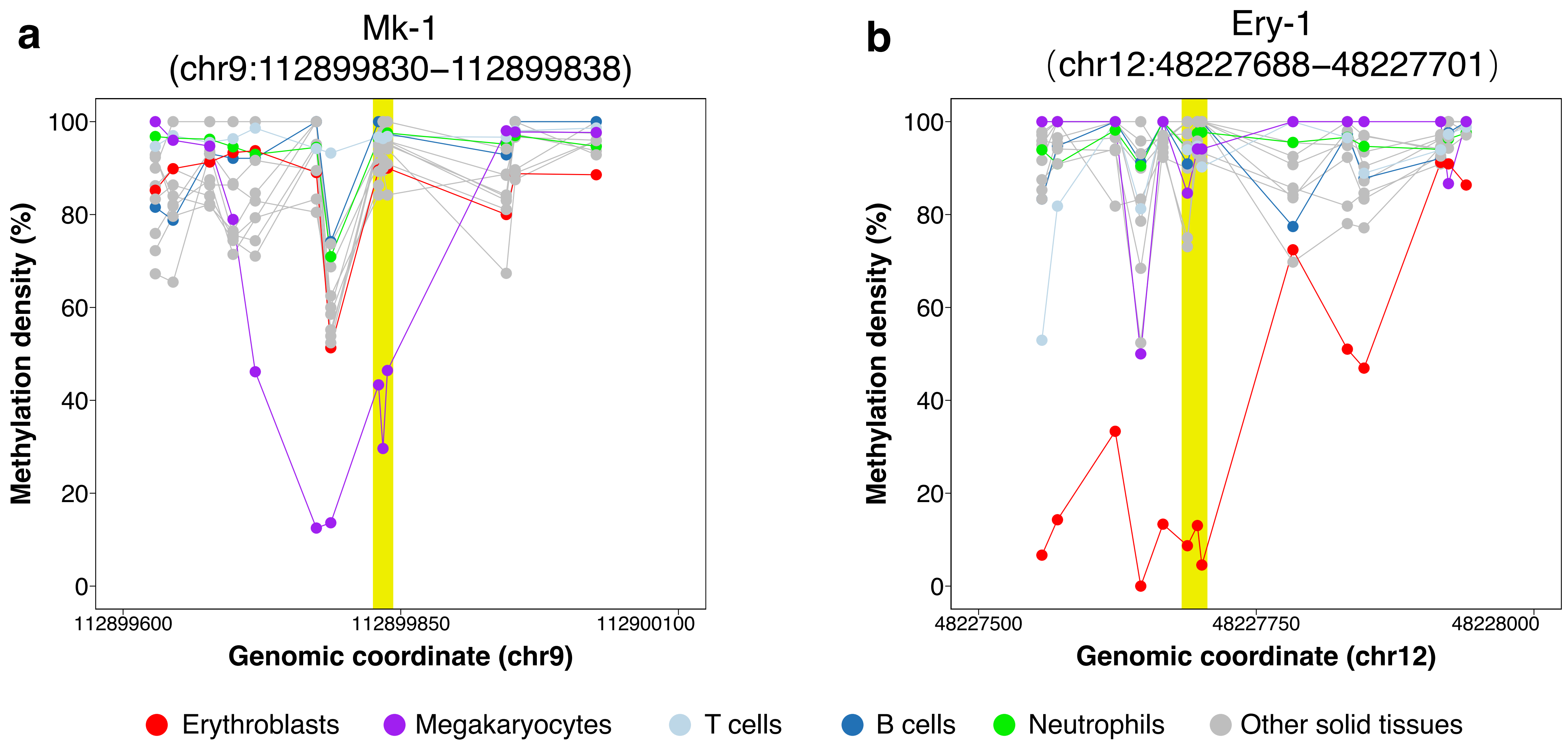

Supplementary Fig. 1. The selected differentially methylated regions (DMRs) of megakaryocytes and erythroblasts. The methylation densities of CpG sites within and around the DMR (shaded in yellow) for erythroblasts (red), megakaryocytes (purple), T cells (light blue), B cells (dark blue), neutrophils (green), and other tissues (grey) were shown. Other tissues include the adipose tissues, adrenal gland, colon, esophagus, liver, lung, pancreas, small intestines.

a Megakaryocyte-specific DMR located in the *PALM2AKAP2* gene on chromosome 9.

b Erythroblast-specific DMR located on chromosome 12.

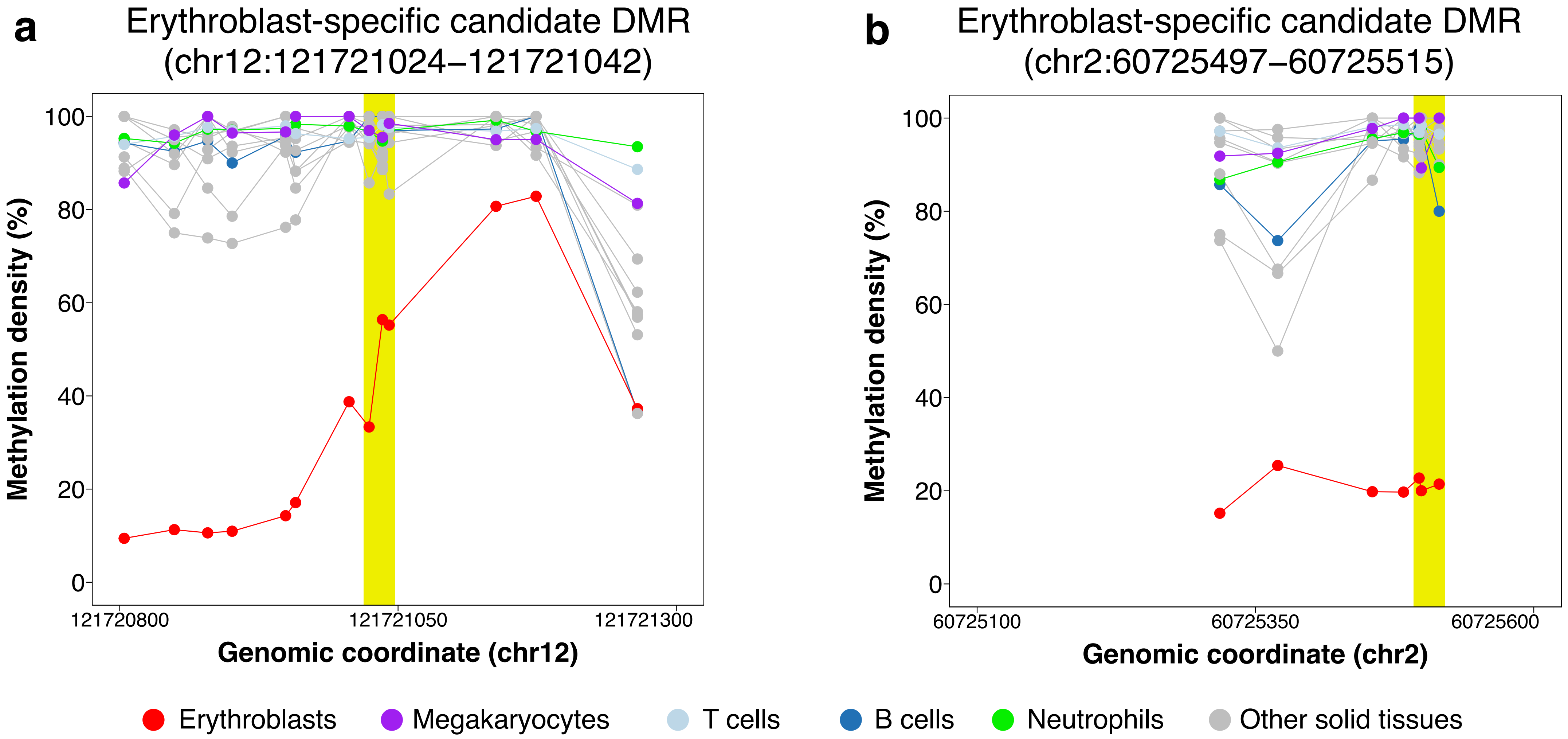

Supplementary Fig. 2. Methylation densities of CpG sites in the other candidate methylation markers. The methylation densities of CpG sites within and around the DMR (shaded in yellow) for erythroblasts (red), megakaryocytes (purple), T cells (light blue), B cells (dark blue), neutrophils (green), and other tissues (grey) were shown. Other tissues include adipose tissues, the adrenal gland, colon, esophagus, liver, lung, pancreas and small intestines.

a A candidate erythroblast-specific marker on chromosome 12

b A candidate erythroblast-specific marker on chromosome 2

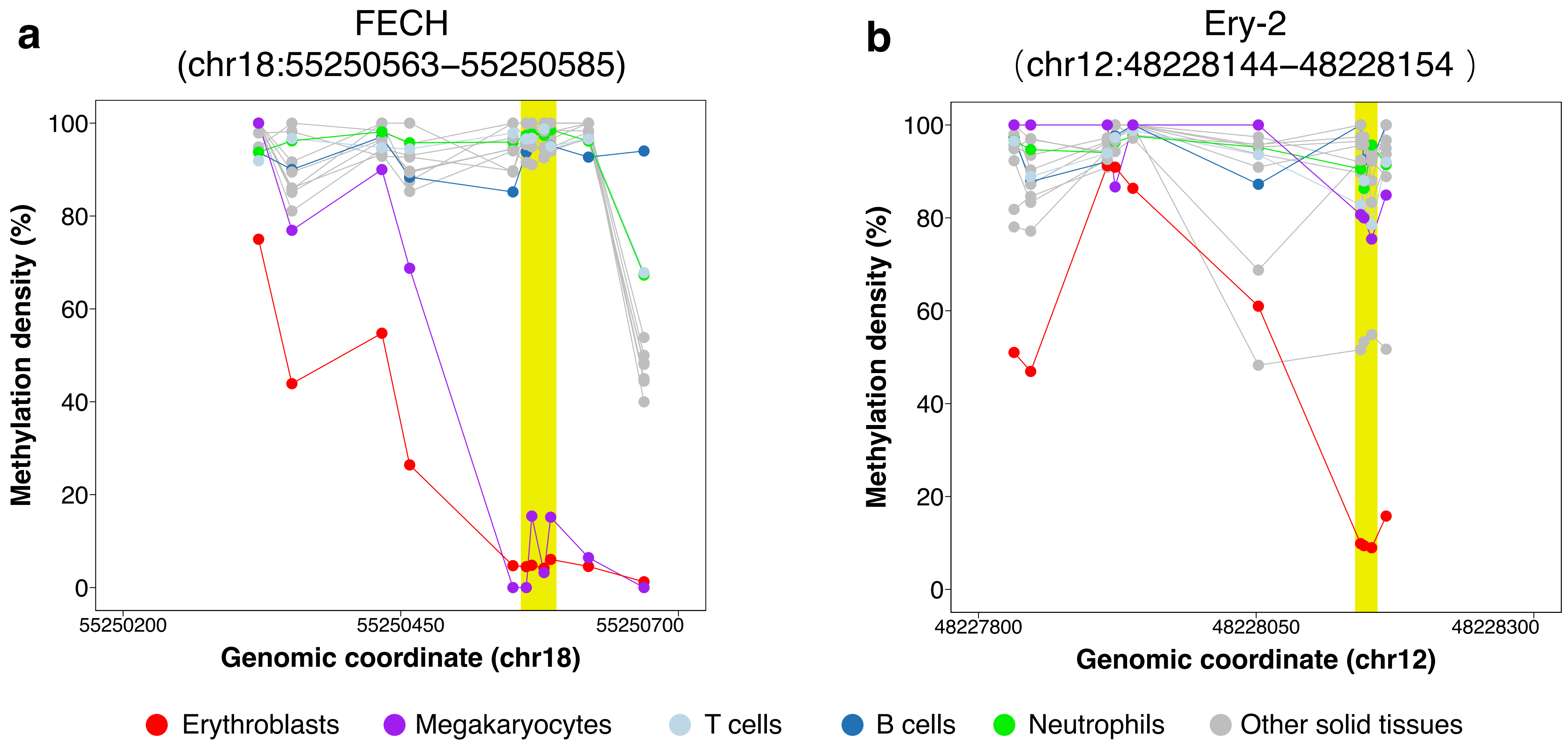

Supplementary Fig. 3. Methylation densities of CpG sites in the previously reported erythroblast-associated methylation markers. The methylation densities of CpG sites within and around the DMR (shaded in yellow) for erythroblasts (red), megakaryocytes (purple), T cells (light blue), B cells (dark blue), neutrophils (green), and other tissues (grey) were shown. Other tissues include adipose tissues, the adrenal gland, colon, esophagus, liver, lung, pancreas and small intestines.

a Ferrochelatase (*FECH*) gene on chromosome 18

b Ery-2 marker located on chromosome 12

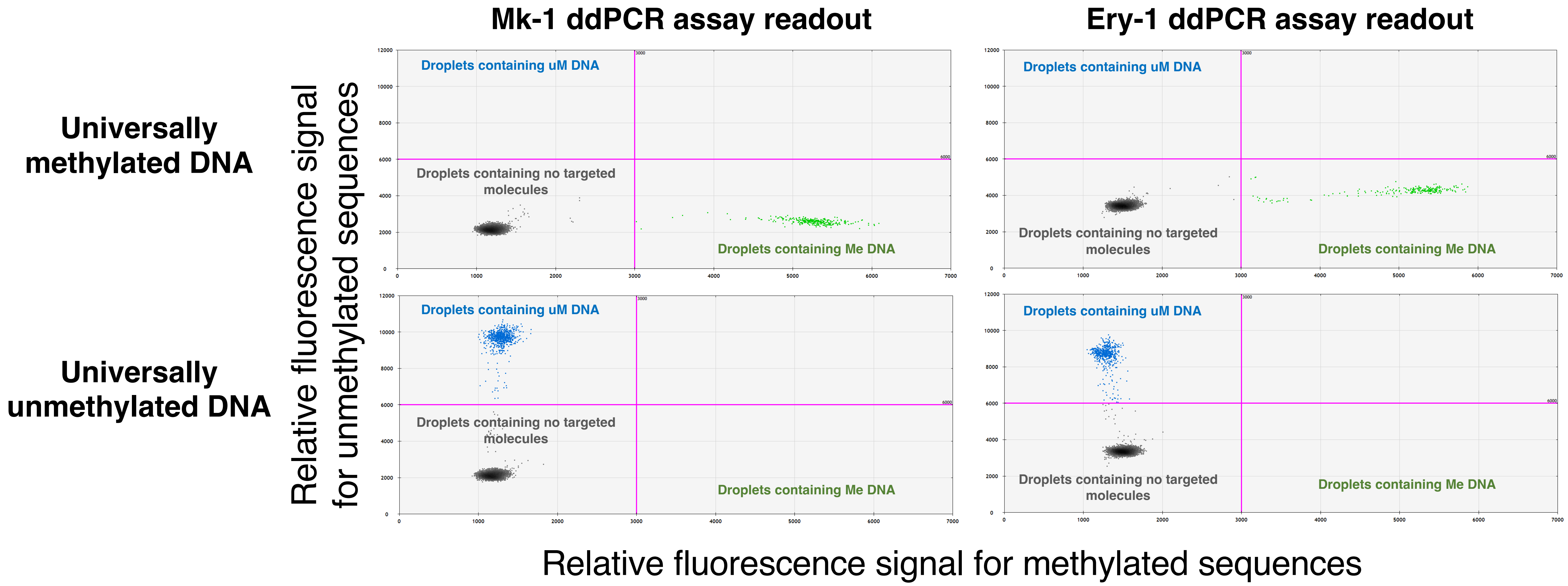

Supplementary Fig. 4. Evaluation of the analytical specificity of the digital PCR assays based on the megakaryocyte-specific (Mk-1) and erythroblast-specific (Ery-1) DMR with the use of fully methylated and fully unmethylated DNA sequences as positive and negative controls.

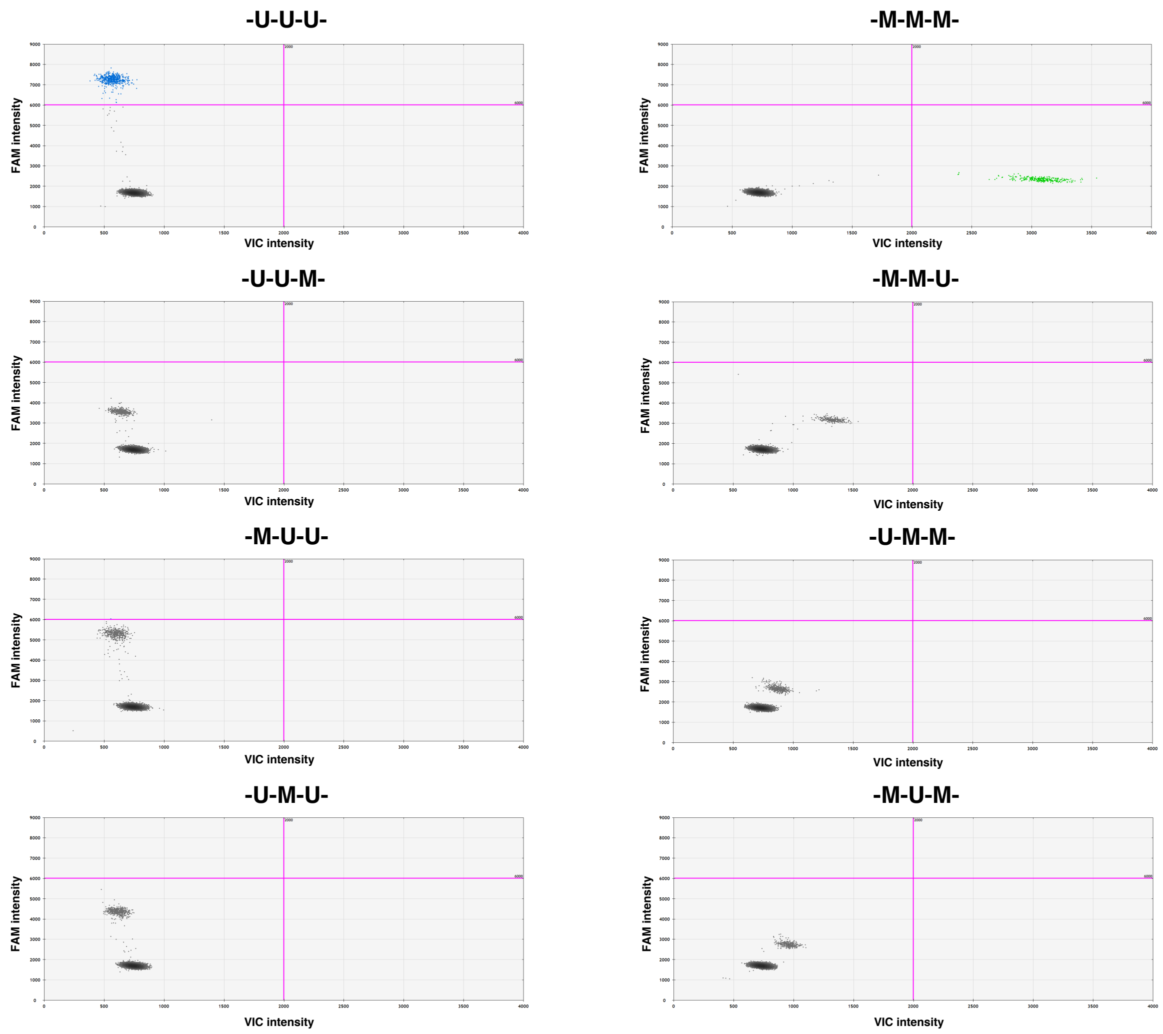

Supplementary Fig. 5. Digital PCR analyses of DNA sequences of the 8 different methylation patterns based on the Mk-1 marker. The unmethylated CpG is denoted as U and the methylated CpG is denoted as M.

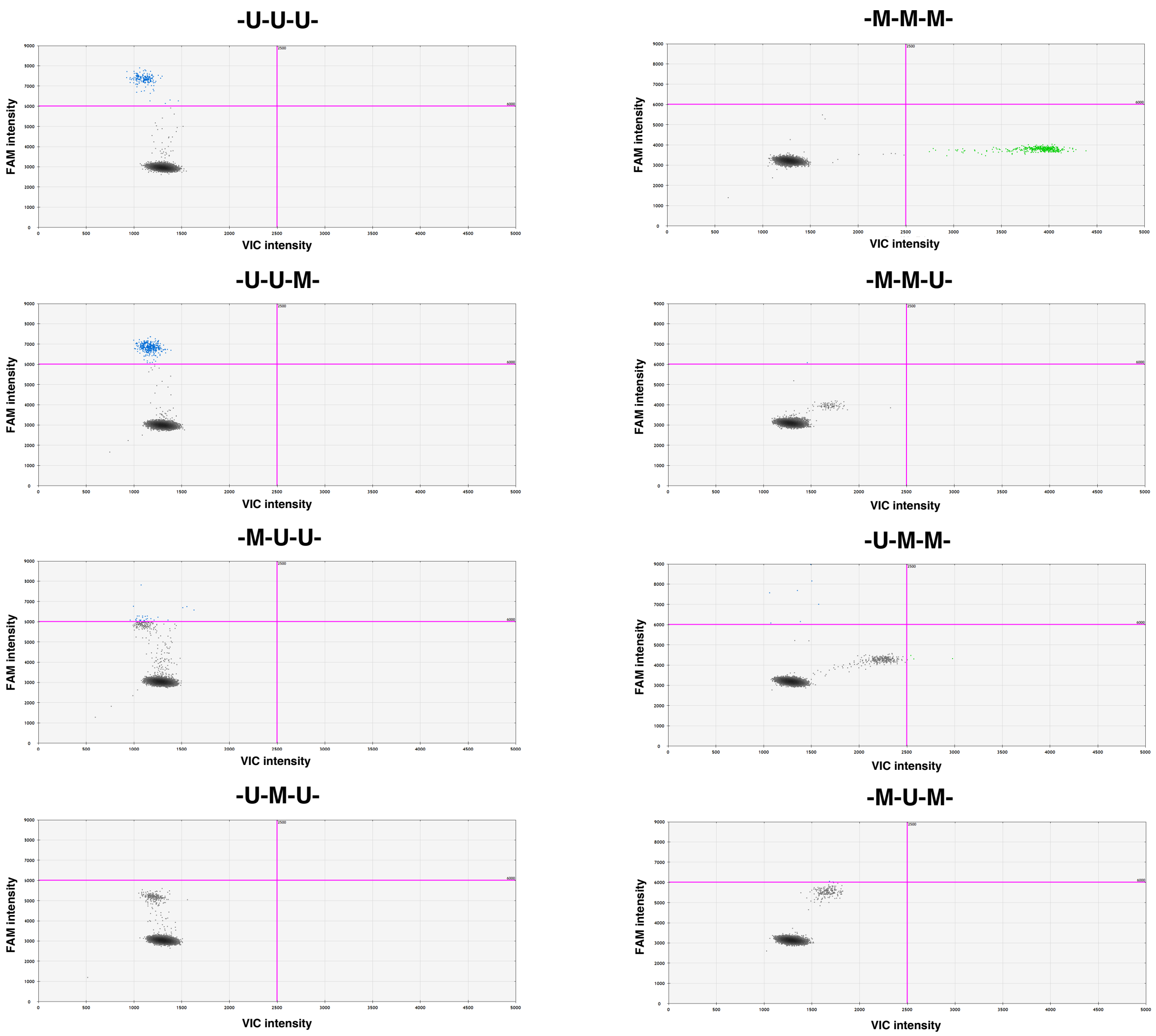

Supplementary Fig. 6. Digital PCR analyses of DNA sequences of the 8 different methylation patterns based on the Ery-1 marker. The unmethylated CpG is denoted as U and the methylated CpG is denoted as M.

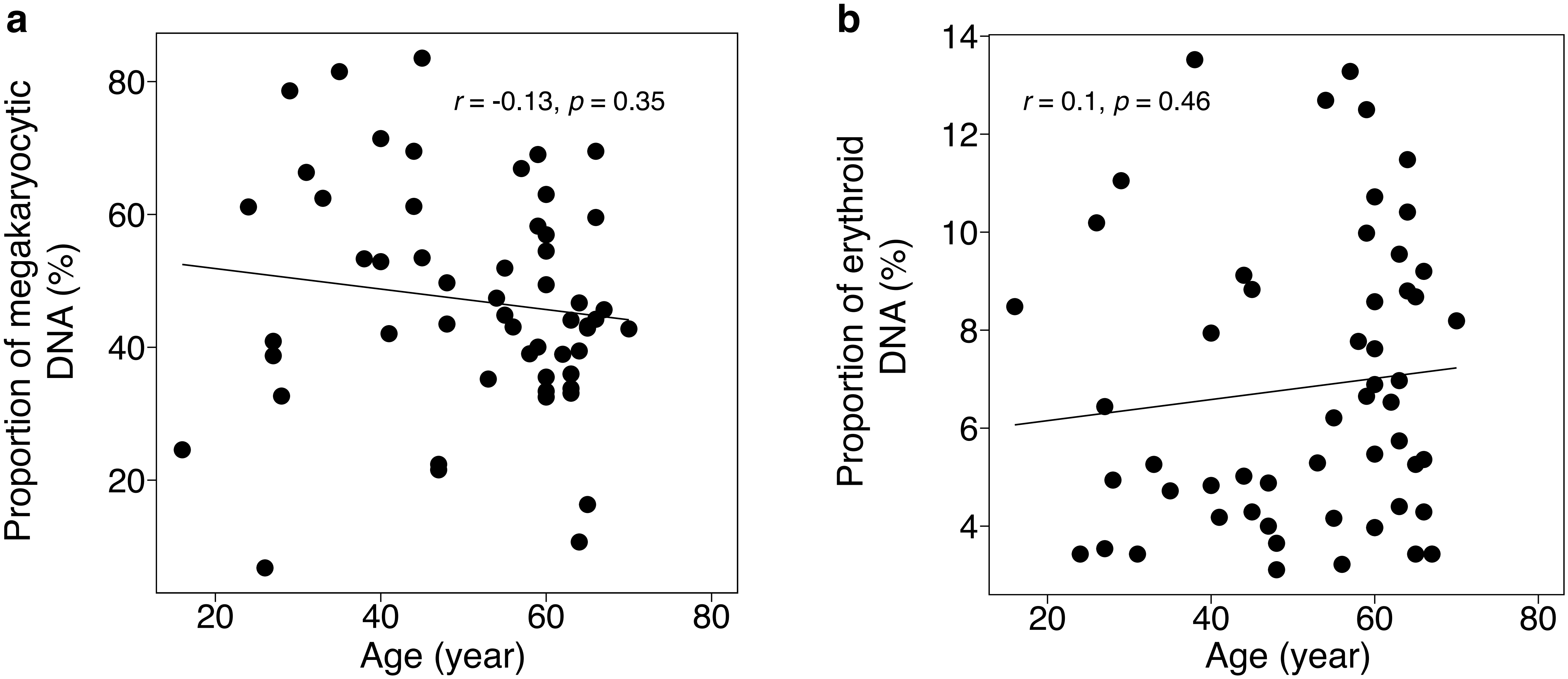

Supplementary Fig. 7. Correlation of age and (a) megakaryocytic DNA proportion and (b) erythroid DNA proportion.

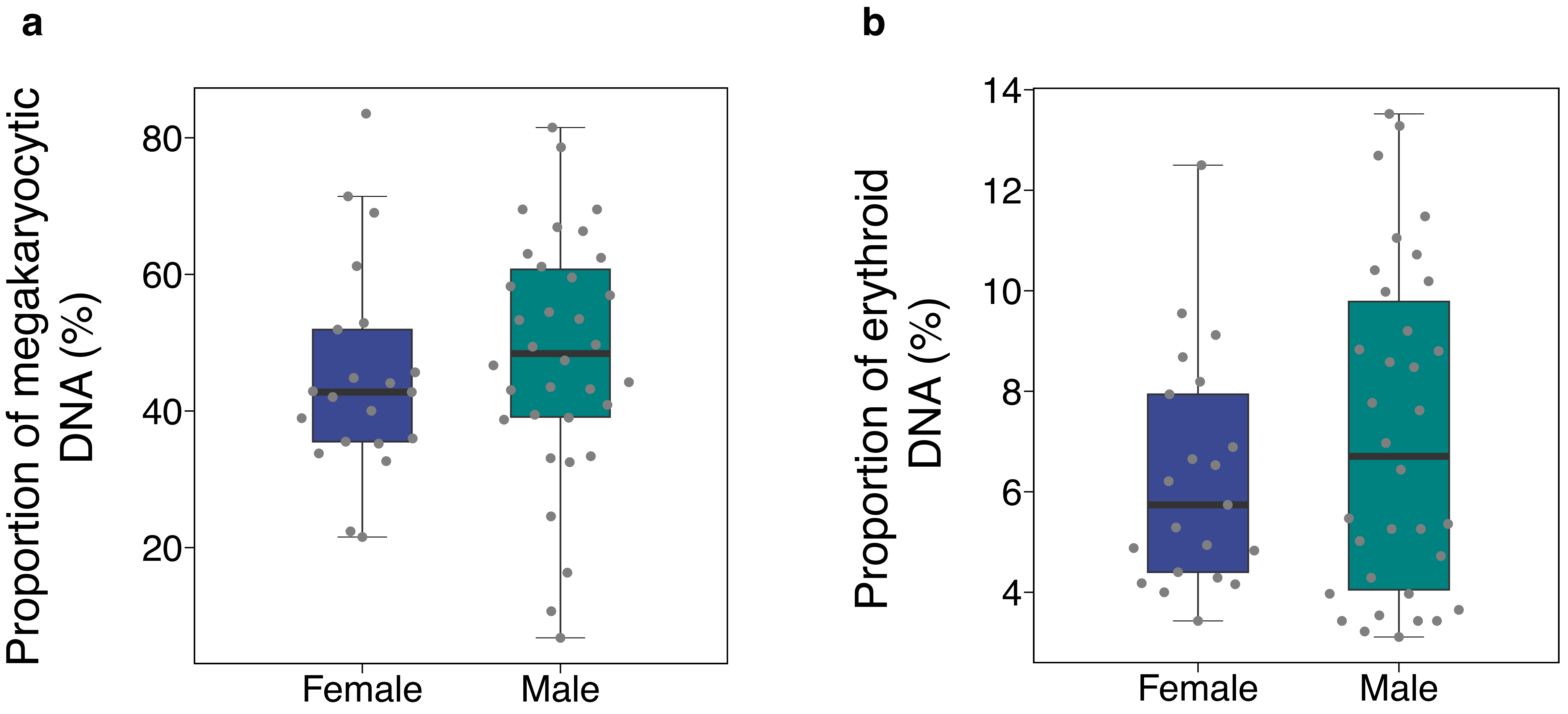

Supplementary Fig. 8. (a) Megakaryocytic DNA proportion and (b) erythroid DNA proportion in male and female healthy control subjects.

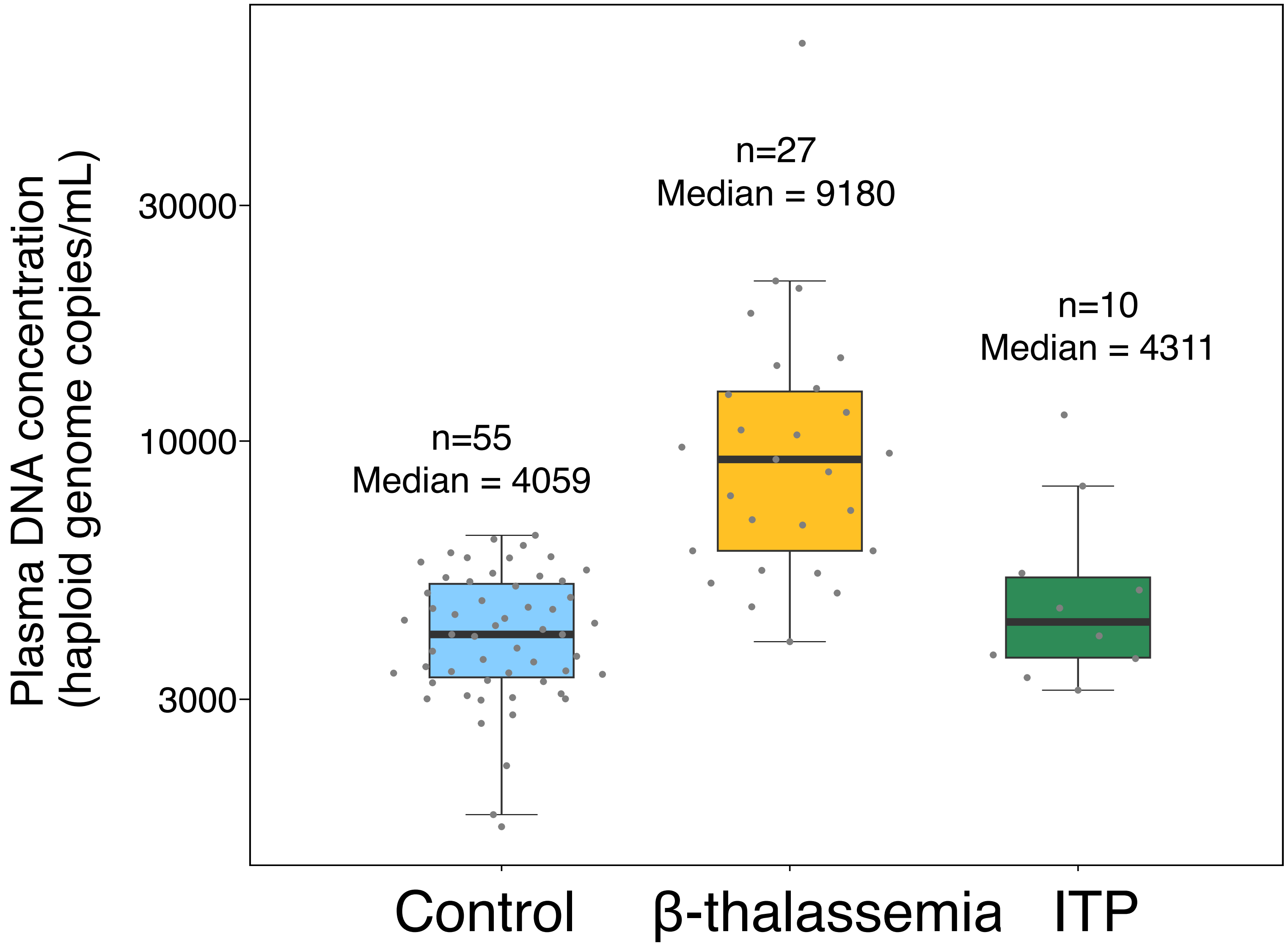

Supplementary Fig. 9. Total plasma DNA concentration (haploid genome copies/mL) in healthy controls, patients with β-thalassemia major and idiopathic thrombocytopenic purpura (ITP).
